# Impairment of central language processing in critically ill coronavirus disease 2019 patients with delirium

**DOI:** 10.1093/braincomms/fcad073

**Published:** 2023-03-25

**Authors:** Fabrice Ferré, William Buffières, Lizette Heine, Beatrice Riu, Jonathan Curot, Alexandra Corneyllie, Benjamine Sarton, Fabien Perrin, Stein Silva

**Affiliations:** Auditory Cognition and Psychoacoustics Team—Lyon Neurosciences Research Center, INSERM U1028—CNRS UMR5292, Le Vinatier Hospital, Bron, France; Critical Care Unit, University Teaching Hospital of Purpan (URM), Toulouse, France; Toulouse NeuroImaging Center (ToNIC) laboratory, UMR INSERM/UPS 1214, University Teaching Hospital of Purpan (URM), Toulouse, France; Critical Care Unit, University Teaching Hospital of Purpan (URM), Toulouse, France; Toulouse NeuroImaging Center (ToNIC) laboratory, UMR INSERM/UPS 1214, University Teaching Hospital of Purpan (URM), Toulouse, France; Auditory Cognition and Psychoacoustics Team—Lyon Neurosciences Research Center, INSERM U1028—CNRS UMR5292, Le Vinatier Hospital, Bron, France; Critical Care Unit, University Teaching Hospital of Purpan (URM), Toulouse, France; Toulouse NeuroImaging Center (ToNIC) laboratory, UMR INSERM/UPS 1214, University Teaching Hospital of Purpan (URM), Toulouse, France; Neurophysiology Department, University Teaching Hospital of Purpan (URM), Toulouse, France; Auditory Cognition and Psychoacoustics Team—Lyon Neurosciences Research Center, INSERM U1028—CNRS UMR5292, Le Vinatier Hospital, Bron, France; Critical Care Unit, University Teaching Hospital of Purpan (URM), Toulouse, France; Toulouse NeuroImaging Center (ToNIC) laboratory, UMR INSERM/UPS 1214, University Teaching Hospital of Purpan (URM), Toulouse, France; Auditory Cognition and Psychoacoustics Team—Lyon Neurosciences Research Center, INSERM U1028—CNRS UMR5292, Le Vinatier Hospital, Bron, France; Critical Care Unit, University Teaching Hospital of Purpan (URM), Toulouse, France; Toulouse NeuroImaging Center (ToNIC) laboratory, UMR INSERM/UPS 1214, University Teaching Hospital of Purpan (URM), Toulouse, France

**Keywords:** delirium, COVID-19, self-processing, semantic priming, event-related potentials

## Abstract

Accumulating evidence indicates that coronavirus disease 2019 is a major cause of delirium. Given the global dimension of the current pandemic and the fact that delirium is a strong predictor of cognitive decline for critically ill patients, this raises concerns regarding the neurological cost of coronavirus disease 2019. Currently, there is a major knowledge gap related to the covert yet potentially incapacitating higher-order cognitive impairment underpinning coronavirus disease 2019 related delirium. The aim of the current study was to analyse the electrophysiological signatures of language processing in coronavirus disease 2019 patients with delirium by using a specifically designed multidimensional auditory event-related potential battery to probe hierarchical cognitive processes, including self-processing (P300) and semantic/lexical priming (N400). Clinical variables and electrophysiological data were prospectively collected in controls subjects (*n* = 14) and in critically ill coronavirus disease 2019 patients with (*n* = 19) and without (*n* = 22) delirium. The time from intensive care unit admission to first clinical sign of delirium was of 8 (3.5–20) days, and the delirium lasted for 7 (4.5–9.5) days. Overall, we have specifically identified in coronavirus disease 2019 patients with delirium, both a preservation of low-level central auditory processing (N100 and P200) and a coherent ensemble of covert higher-order cognitive dysfunctions encompassing self-related processing (P300) and sematic/lexical language priming (N400) (spatial–temporal clustering, *P*-cluster ≤ 0.05). We suggest that our results shed new light on the neuropsychological underpinnings of coronavirus disease 2019 related delirium, and may constitute a valuable method for patient’s bedside diagnosis and monitoring in this clinically challenging setting.

## Teaser

Ferré *et al*. report covert high order cognitive dysfunctions, encompassing prediction of lexical/semantic occurrence and self-relevant auditory stimuli detection, in delirium versus non-delirium critically ill coronavirus disease 2019 patients by means of electrophysiological signatures (N400 and P300 event-related potentials).

## Graphical Abstract

**Graphical Abstract:**
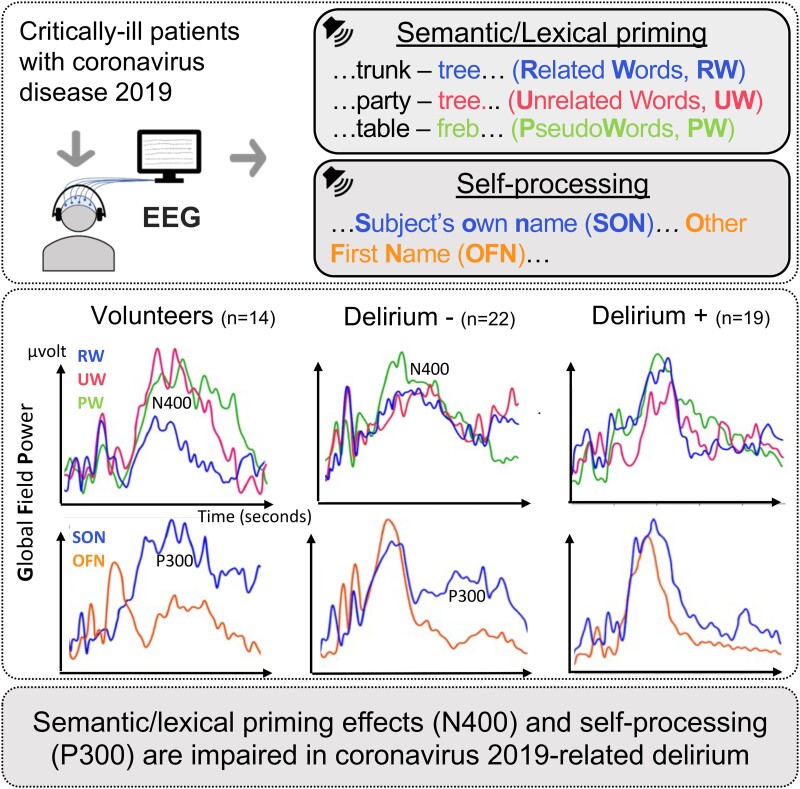

## Introduction

Delirium, defined as an acute disorder of attention and cognition,^1^ is a ubiquitous manifestation of acute brain dysfunction and is well known to be a dangerous untoward prognostic development for intensive care unit (ICU) patients.^2^ Delirium is an independent predictor of longer time in ICU, mortality and cost.^3,4^ Moreover, convergent data suggest that delirium is a strong predictor of cognitive decline that persists for months to years after ICU stay.^5^

Emerging evidence indicates that the severe acute respiratory syndrome coronavirus 2 (SARS-CoV-2), the etiologic agent of coronavirus disease 2019 (COVID-19), is a major cause of delirium affecting 55 to 84% of critically ill COVID-19 patients.^6-9^ Given the global dimension of the current pandemic and the high frequency of delirium among severe COVID-19 patients, this raises concerns regarding the neurological burden of COVID-19.^9^ Recent anatomopathological evidence suggest that these processes are responsible of widespread brain damage, mainly in brainstem and major associative cortices structures.^8,10^ However, there is no clear *in vivo* evidence about the cognitive impairments that are associated to these reported brain injuries. Hence, there is an urgent need for studies aiming to provide a fine-grained description of the extent of cognitive dysfunctions that are related to COVID-19 delirium, to help foster research that will ultimately lead to its prevention and treatment.

Converging data have demonstrated the ability of auditory event-related potentials (ERPs) to probe a large ensemble of covert low- and high-level cognitive processes at the bedside of patients with disorders of consciousness.^11,12^ Indeed, it is well established that auditory ERPs allow an accurate assessment of hierarchically organized cognitive processes including auditory perception, attentional focus, stimuli memorization, self-processing and detection of semantic discordances.^13-16^

Given the reports of specific cortical associative lesions (i.e. frontal and temporal lobes) in COVID-19 patients with delirium,^17,18^ we hypothesize that COVID-19 related delirium is underpinned by a significant impairment of high-level cognitive processes that sustain self-processing and awareness. We suggest that these cognitive dysfunctions can be accurately assessed through the study of electrophysiological markers of both auditory discrimination of self-relevant words and prediction of semantic or lexical occurrence. Hence, the main aim of the current study was to analyse the electrophysiological signatures of central language processing in COVID-19 patients with delirium by using a specifically designed multidimensional auditory ERP battery. As a secondary aim and seeking to provide a control group, we also studied with the same methods, the cognitive impact of SARS-CoV-2 infection in patients who were critically ill and conscious, but without delirium.

## Methods

### Design and population

**Figure 1:**
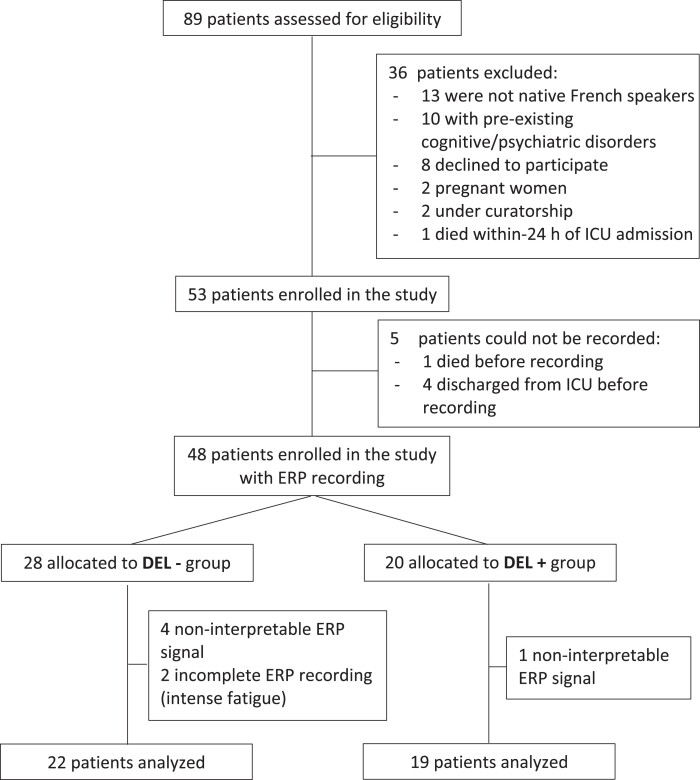
**Study flowchart.** ICU, intensive care unit; ERP, event-related potentials; DEL−, COVID-19 patients without delirium; DEL+, COVID-19 patients with delirium; CAM-ICU, confusion assessment for the ICU.

The whole dataset was prospectively collected (Critical Care Unit, University Hospital Purpan, Toulouse, France) between March 2021 and October 2021 (Fig. 1). After a complete withdrawal of any sedative agent, patients were screened for delirium daily over the whole ICU stay [Confusion Assessment Method for the Intensive Care Unit (CAM-ICU) score] by trained ICU medical staff members. COVID-19 diagnoses were confirmed by positive real-time reverse transcription polymerase-chain-reaction (RT-PCR) assay for pharyngeal swap specimens. Inclusion criteria were adults who were critically ill patients between 18 and 80 years of age with COVID-19. Exclusion criteria were past medical history of psychiatric disorders, preexisting significant cognitive deficits [short Informant Questionnaire on Cognitive Decline in the Elderly (short IQCODE) ≥3.6],^19^ blindness, deafness, non-French speakers and pregnancy. The study was approved by the ethics committee of the University Hospital of Toulouse, Toulouse, France (Ref. RC 31/20/0441); and written consent was obtained from all patients.

### Behavioural assessment

In case of delirium, delirium severity (CAM-ICU-7)^20^ was scored on the day of ERPs recording. According to current guidelines,^21^ the level of arousal of patients with delirium was assessed using the Richmond agitation–sedation scale (RASS) to allow identification of hypoactive, hyperactive or mixed delirium phenotypes.^22^ Delirium and coma duration were also collected, and the number of days with acute brain dysfunction was defined as the number of days spent in coma and/or delirium.

### Auditory stimulation paradigms

**Figure 2:**
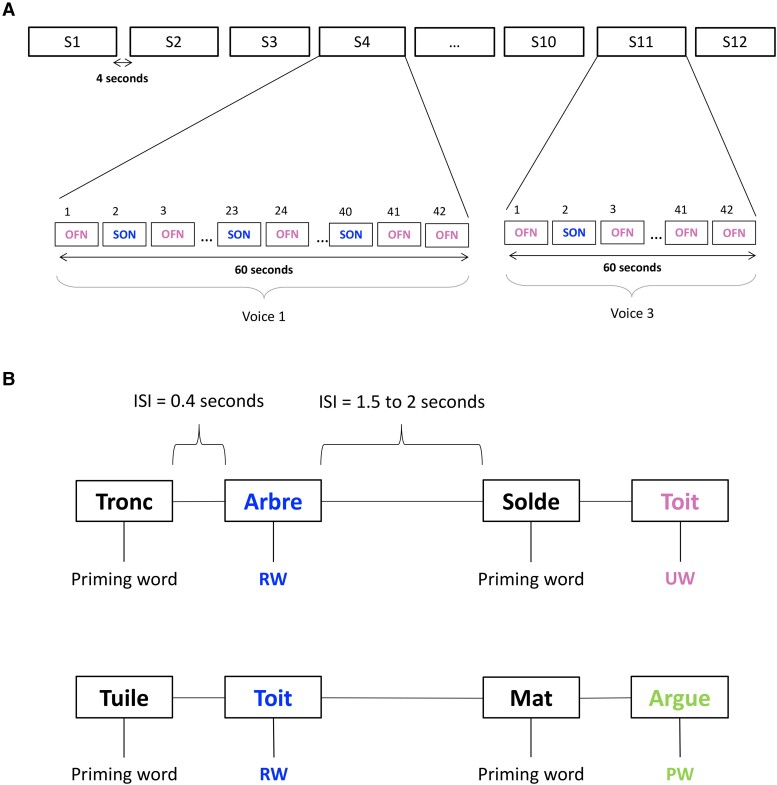
**Multidimensional auditory battery.** (**A**) Subject's own name (SON) paradigm. For each subject, 12 sequences (S) of 42 names (1 subject's own name (SON) + 6 other unfamiliar first names (OFNs) were presented 6 times in a pseudo-random order. All first names were presented with the same probability (1/7 ≈ 14.3% for each name). The inter-stimulus interval (ISI) ranged from 400 to 500 msec. Each sequence was spoken with one voice randomly selected from three. The duration of a sequence of 42 names was ∼60 sec. Each sequence was separated by 4 sec. The duration of this experiment was ∼12 minutes. (**B**) Semantic and lexical priming (SLP) paradigm. Eighty pairs of semantically related (congruent) words (RW), 80 pairs of semantically unrelated words (UW) and 160 pairs of word-pseudoword (PW) were created. The whole protocol consisted of 1 block containing all stimuli distributed in a pseudo-random order and avoiding more than three successive repetitions of the same category of pairs. The inter-stimulus interval (ISI) between words in a pair was fixed at 400 msec. The ISI between pairs was variable and lasted from 1500 to 2000 msec, in random steps of 100 msec. A 60 sec pause was introduced in the middle of the protocol. The duration of this experiment was ∼21 minutes.

A specifically designed auditory ERP battery was used (Fig. 2 and Supplementary Text). This battery encompasses two independent ensembles of auditory stimuli: the subject’s own name (SON) and the semantic and lexical priming (SLP) paradigms. During the SON paradigm, seven first names were selected for each subject: the subject’s own first name (SON) and six other unfamiliar first names (OFN). The SLP paradigm was based on pairs of items including congruent related words (RW) and incongruent unrelated words (UW) or pseudoword (PW). This such design allows the study of both semantic (RW vs UW) and lexical (RW vs PW) priming effects. The order of SON and SLP paradigms was randomized. All auditory stimuli were individually created using NaturalReader 14 (16 bits, 44 100 Hz), equalized to the same dB-A-weighting level (around 65 dB-A) and presented binaurally during the experiment using a Raspberry Pi.^23^

### Electrophysiological recordings

High-density EEG data was recorded during auditory stimulation using a sampling rate of 500 Hz with a 128-electrode geodesic sensor net (EGI®, Philips) referenced to the vertex (Cz). Data was first visually inspected to identify bad channels. Data were bandpass filtered between 0.5 and 25 Hz using a zero-double filter and a notch at 50 Hz. Eye channels were recreated through subtraction of the channels above and below the eye, an average reference was taken and Cz was interpolated using the spherical spline method implemented. For any subject where data was affected by eye-blinks, an Independent Component Analysis (fastICA) was performed to remove the blink components from the signal. Trials were then segmented (epochs) from −200 msec to +1000 msec relative to the onset of the stimulus, and a baseline correction (−200 to 0 msec) was applied. To further clean the data, an automatic rejection function was used where bad trials were either interpolated or rejected based on trial-wise assessment of individual sensor thresholds.^24^ All those processing stages were performed using Python version 19.2.

### Statistical analysis

For both auditory paradigms, averages of the cerebral response to targets were computed for each individual. Effects between stimuli: SON (SON vs OFN), semantic (RW vs UW) and lexical (RW vs PW) were tested at the group level for healthy participants, COVID-19 patients with and without delirium (DEL+ and DEL−, respectively). Global field power (GFP), which is the root summed square of the voltage of all electrodes at each time point^25^ and which GFP provides a compact summary of the time course of the cerebral response to a stimulus, was also and can be computed at the group level for each stimuli and paradigm by averaging the individual GFP.

To test statistical differences between stimuli (SON vs OFN, RW vs UW/PW) at the group level, we used spatio-temporal clustering permutation tests with one sided *t*-tests and 1000 permutations.^26^ Cluster level alpha was set to 0.01 with a cluster forming threshold of 0.05 and 0.1.

### Data availability

All data and codes are available upon request to the corresponding author. Codes used in the study are available at: https://github.com/crnl-lab/EEG_2021_CAP_FPerrin_ComaDelirium_FFerre.

## Results

A total of 89 COVID-19 critically ill patients were prospectively assessed for eligibility, and 48 of them were enrolled in the study (Fig. 1). Their characteristics (demography, ICU severity and ongoing treatments) are available in Supplementary Table 1. Five patients had to be excluded because of insufficient electrophysiological data quality, and 2 patients withdrew consent. The final cohort consisted of 41 patients, age 65.0 (55 to 71.5) years, of whom 42 (87.5%) were males (Supplementary Table 1). Nineteen of them experienced delirium. The time from ICU admission to first clinical sign of delirium was of 8 (3.5–20) days and the delirium lasted for 7 (4.5–9.5) days (Supplementary Table 2). Clinical and electrophysiological data were also collected in controls subjects (*n* = 14).

### Subject’s own name

**Figure 3:**
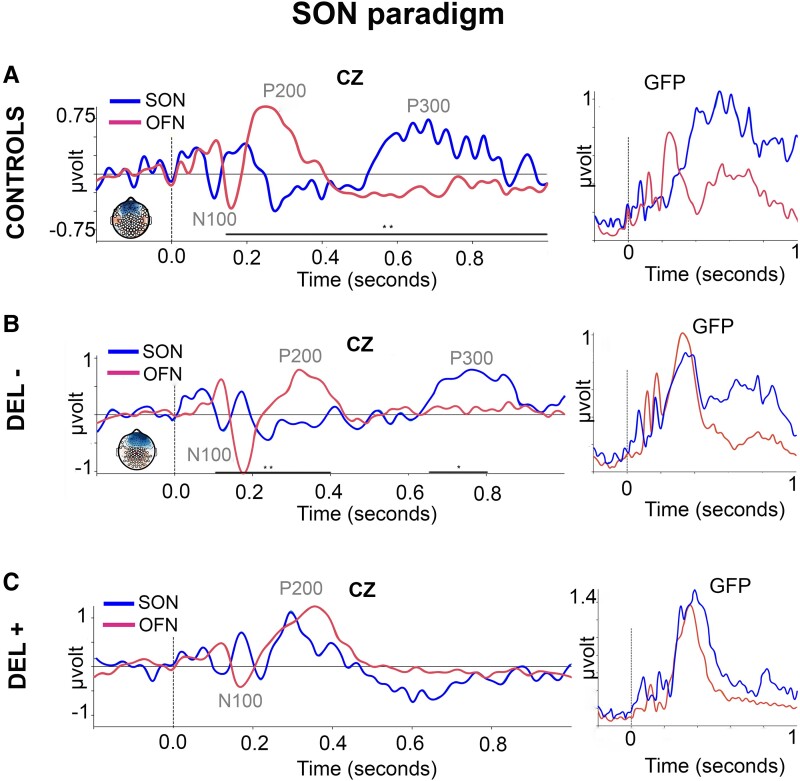
**Subject’s own name (SON) paradigm.** Event-related potentials (ERPs) at Cz and global field power (GFP) in 14 healthy participants [CONTROLS (**A**)], 22 COVID-19 patients without delirium [DEL− (**B**)] and 19 COVID-19 patients with delirium [DEL+ (**C**)] in response to subject’s own name (SON, blue curves) and to other first names (OFN, magenta curves). Spatio-temporal clustering permutation tests with one sided *t*-tests and 1000 permutations. Significance threshold: alpha cluster was set to 0.01; **P ≤ 0.05 and *P ≤ 0.1 at each sample for SON and OFN comparison.

ERPs on Cz and GFP at the group level in response to SON paradigm are reported in Fig. 3. Individual electrophysiological data is provided in Supplementary Table 3. In all patients, either with (*n* = 19) and or without delirium (*n* = 22) and controls subjects (*n* = 14), an early negative electrical event centred on the vertex (Cz) corresponding to a typical N100 component was observed. Moreover, in healthy participants, a P200 component to OFN and a P300 component to SON conditions were clearly identified at a group level (Fig. 3A). The difference between OFN and SON conditions was statistically significant for this group and its topography corresponded to a centro-parietal cluster (*P* ≤ 0.05 at each sample).

In the case of patients of patients without delirium (Fig. 3B), the ERPs components elicited by the OFN and SON conditions began respectively at 200 and 600 msec after stimulus onset. As in the control group, A P300 effect to SON was observed in these critically ill COVID-19 patients without delirium at a group level and corresponded to a significant centro-parietal cluster (*P* ≤ 0.1 at each sample).

In COVID-19 patients with delirium, there was no statistically significant difference between the ERPs generated during the OFN and SON conditions (Fig. 3C).

### Semantic and lexical priming

**Figure 4:**
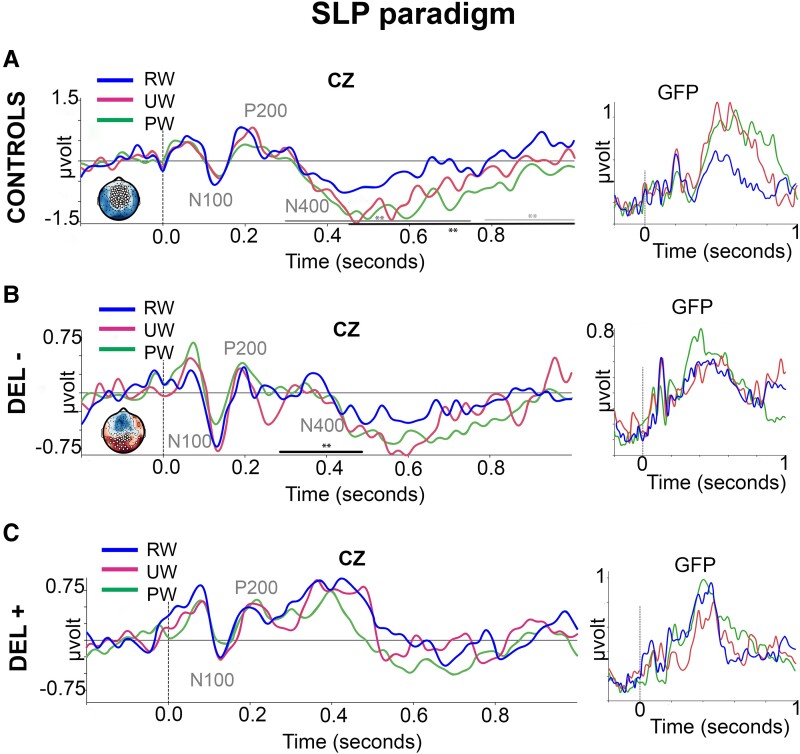
**Semantic and lexical priming (SLP) paradigm.** Event-related potentials (ERPs) at Cz and global field power (GFP) in 14 healthy participants [CONTROLS (**A**)], 22 COVID-19 patients without delirium [DEL− (**B**)] and 19 COVID-19 patients with delirium [DEL+ (**C**)] in response to semantically related words (RW, blue curves), unrelated words (UW, magenta curves) and to lexically unrelated pseudo-words (PW, green curves). Spatio-temporal clustering permutation tests with one sided *t*-tests and 1000 permutations. Significance threshold: alpha cluster was set to 0.01; black **P ≤ 0.05 for RW and PW comparison and dark grey **P ≤ 0.05 for RW and UW comparison and light grey **P ≤ 0.05 for UW and PW comparison at each sample and *P ≤ 0.1 for RW and PW comparison at each sample.

ERPs on Cz and GFP at the group level in response to SLP paradigm are reported in Fig. 4. Individuals ERPs data is reported in Supplementary Table 3. In controls subjects (*n* = 14) and in all critically ill COVID-19 patients, either with (*n* = 22) and or without delirium (*n* = 19) in both controls and all critically COVID-19 patients (with and without delirium), we observed two successive early electrical events centred on the vertex (Cz), corresponding to typical N100 and P200 components. Semantic (UW vs RW) and lexical (PW vs RW) effects were clearly observed in controls subjects (*P* ≤ 0.05 at each sample). These effects began ∼400 msec after stimulus (Fig. 4A) and had a fronto-central topography. A significant N400 component was also observed in COVID-19 patients without delirium (Fig. 4B), in particular, during the lexical condition (PW vs RW). Indeed, a parieto-occipital cluster of significant effects was observed after PW vs RW stimulus onset (*P* ≤ 0.1 at each sample). Remarkably, both semantic (UW vs RW) and lexical (PW vs RW) effects were abolished in COVID-19 patients with delirium, as only overlapping peaks with slight slopes were identified across all conditions (UW, RW and PW) (Fig. 4C).

**Figure 5:**
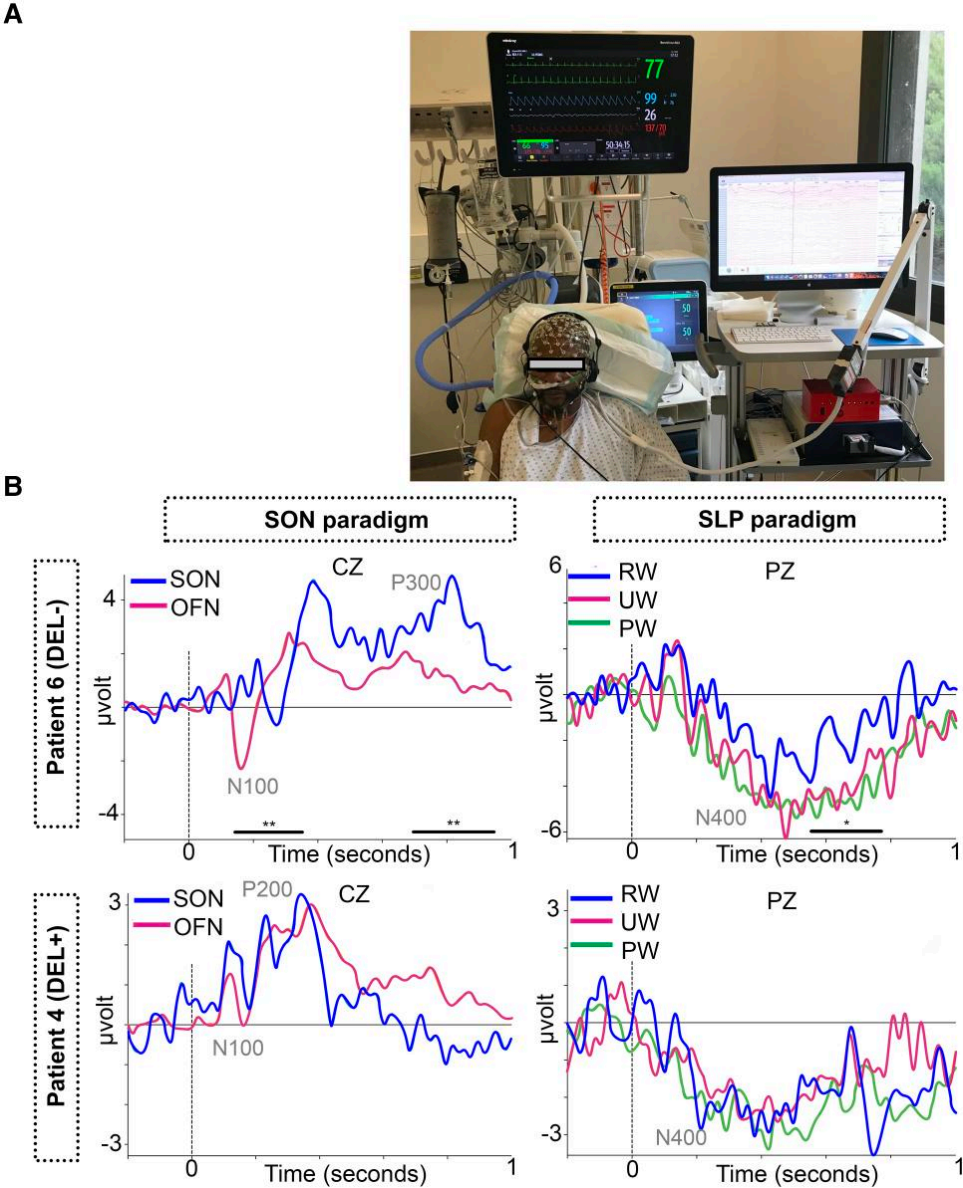
**Illustrative cases.** Multidimensional cognitive electrophysiological assessment in critically ill COVID-19 patients in ICU (**A**). ERPs from two patients are represented, one COVID-19 patient without delirium (DEL−, patient 6) and one COVID-19 patient with delirium (DEL+, patient 4). Spatio-temporal clustering permutation tests with one sided *t*-tests and 1000 permutations. Significance threshold: alpha cluster was set to 0.01; **P ≤ 0.05 for SON and OFN comparison at each sample and *P ≤ 0.1 for RW and PW comparison at each sample. SON, subject’s own name; SLP, semantic and lexical priming; RW, related words; UW, unrelated words; PW, pseudo-words.

Overall, semantic (RW vs UW) and lexical (RW vs PW) N400 effects were observed in the group of healthy participants. Lexical but not semantic N400 effects were observed in COVID-19 patients without delirium. Both of these N400 linguistic effects were absent in COVID-19 patients with delirium. Illustrative cases of COVID-19 patients are reported in Fig. 5. No specific profile was observed between standard EEG and auditory ERPs data (Supplementary Table 4).

No correlation was found between ERPs preservation/abolition and ICU or hospital lengths of stay (Supplementary Table 5).

## Discussion

Delirium can occur in any patient in ICU but accumulating data suggest that we are seeing more than expected and that these symptoms could be characteristics of SARS-CoV-2 infection.^27^ Critically, COVID-19 related delirium poses the problems of potential neurological sequalae and the risk of overwhelming an already fractured health-care system.^28^ Consequently, we urgently need to understand and respond to potentially incapacitating neurological consequences of COVID-19 in the acute phases of the disease. In this context, we reported new evidence about COVID-19 patients with delirium covert inabilities to discriminate self-relevant words and to predict semantic or lexical occurrence. It is worth noting that these electrophysiological signatures were not observed in critically ill COVID-19 patients without delirium.

Firstly, we observed that compared to controls subjects, the first canonical levels of central auditory processing were preserved in all COVID-19 patients, independently of the diagnosis of delirium. Indeed, early auditory ERPs were identified in all patient’s groups within the expected temporal integration window (N100 and P200). We suggest that this result is in line with recent reports on the preservation of perceptual auditory performance—including the encoding of spectrotemporal characteristics of sounds—by COVID-19 patients, even in the case of anosmia.^29^

Secondly, we identified that COVID-19 patients with delirium, compared to controls and COVID-19 patients without delirium, had a significant impairment of their ability to categorize self-related stimuli. Converging electrophysiological studies have demonstrated that P300 evoked potential by hearing one’s own first name, presented equiprobably within other unfamiliar first names, is modulated by stimuli salience and subject’s self-related awareness.^16^ Interestingly, many studies have reported the usefulness of SON P300 to probe self-awareness impairment both in patients with chronic disorders of consciousness^11^ or psychotic disorders, as schizophrenia.^15^ Theoretical frameworks pose that P300 anomalies are related to either a memory storage deficit or closure cognitive epoch anomaly.^11,15,30^ Moreover, combined electrophysiological and neuroimaging studies suggest that P300 identified impairments to treat self-relevant auditory stimuli, might be the consequence of frontal lobes dysfunction.^30^

Finally, we report new evidence about a significant impairment of COVID-19 patients with delirium to use lexical and semantic memories to anticipate the processing of linguistic stimuli. We think that the reported evidence of COVID-19 patients with delirium inability to benefit from a semantic priming can be a related to either a dysfunctional attentional spotlight or an inadequate working memory storage.^31^ It should be noted that N400 abolitions have been described in alternative clinical models of impairments of higher-order cognitive processes, as chronic disorders of consciousness, psychotic disorders and Alzheimer’s disease.^13^

Only scarce data exist on language auditory processing in COVID-19 patients. A recent report of 17 critically ill COVID patients based on brainstem auditive evoked potentials (BAEP) was in favour of a preservation of first-order central auditory functions.^32^ To the extent of our knowledge, we report for the first-time electrophysiological evidence of significant higher-order central language processing dysfunctions in critically ill COVID-19 patients with delirium, notwithstanding we observed a similar preservation of lower-order central auditory processing. First, we have identified in critically ill COVID-19 patients with delirium both a preservation of low-level central auditory processing and a coherent ensemble of higher-order cognitive dysfunctions encompassing self-related processing and sematic/lexical language priming. It can be hypothesized that taking together these anomalies, encompassing higher-order cognitive dysfunctions encompassing self-related processing and sematic/lexical language priming, can be surrogates electrophysiological markers of COVID-19 related brain damages in associative cortices, specifically in temporal and frontal lobes.^18^ This neuropsychological hypothesis is well supported by evidence from neuropathological and molecular findings of patients with SARS-CoV-2 infection who died and underwent autopsy.^27^ Moreover, accumulating evidence from MRI, ^18^F-FDG-PET and EEG support the implication of temporal and frontal lobes dysfunction in COVID-19 patients.^28^

Furthermore, besides the coherent ensemble of higher-order cognitive dysfunctions encompassing self-related processing (P300) and sematic/lexical language priming (N400) that we have identified in patients with delirium, we have also observed a specific abolition of semantic priming in critically COVID-19 patients without delirium. Based on theoretical frameworks of central language processing,^31^ we hypothesize that this specific higher-order cognitive dysfunction might be related to patient’s dysfunction of either attention or working memory capacities. It should be noted that both these mechanisms are in line with previously discussed COVID-19 related brain associative cortices.^18^

A growing body of evidence suggest that innovation in neuroimaging and electrophysiologic techniques now may permit detection of covert key cognitive processes not readily discernable by ICU patient’s bedside examination.^33^ We suggest that our findings are a new paving stone in this research pathway and hold the promise to provide robust and valuable methods to transfer this knowledge from bench to COVID-19 patient’s bedside, to ultimately allow future longitudinal studies specifically designed to determine the acute, mid-, and long-term COVID-19 effects in the central nervous system.

Our results must be interpreted with caution, and a number of limitations should be borne in mind. The first is related to the limited sample size. Consequently, the reported evidence requires confirmation from large-scale trials with strict recruitment criteria. As a second limitation, we acknowledge that we cannot conclude that the observed electrophysiological signatures represent either a specific feature of COVID-19 associated delirium or just a marker of ICU-delirium. Future studies should focus on data analysis from ICU patients with diverse severity of delirium and without SARS-CoV-2 infection to further investigate this issue. As a third limitation, we acknowledge that several recordings were discarded because of poor quality. It is worth noting that our proportion of poor-quality data is similar to previously reported in the context of chronic disorders of consciousness.^12^

Given the global dimension of the current pandemic and the high frequency of delirium among severe COVID-19 patients, there is concern regarding the long-lasting deleterious neurocognitive consequences of COVID-19 which may be dramatic and widespread in the population. We report that COVID-19 related delirium is associated to a large span of higher-order cognitive anomalies, encompassing both self-processing and semantic/lexical priming anomalies. We suggest that our results contribute to fill the knowledge gap related to the covert yet potentially incapacitating consequences of COVID-19 and can significantly contribute to the patient’s bedside detection, monitoring and prognostication of the underlying major cognitive dysfunctions.

## Supplementary Material

fcad073_Supplementary_Data

## Abbreviations

CAM-ICU =: Confusion Assessment Method for the Intensive Care Unit
CNS =: central nervous system
COVID-19 =: novel coronavirus infectious disease 2019
ERP =: event-related potential
ICU =: intensive care unit
IQCODE =: Informant Questionnaire on Cognitive Decline in the Elderly
RASS =: Richmond agitation–sedation scale
SARS-CoV-2 =: severe acute respiratory syndrome coronavirus 2
SLP =: semantic and lexical priming
SON =: subject’s own name
