# Supplementary material for: Impairment of central language processing in critically ill coronavirus disease 2019 patients with delirium
Source: Brain Commun. 2023 Mar 25;5(2):fcad073. doi: 10.1093/braincomms/fcad073 (PMC10066571; doi:10.1093/braincomms/fcad073)
Supplement: fcad073_Supplementary_Data [file fcad073_supplementary_data.docx]

*Supplementary Text.* **Multidimensional auditory paradigms battery.** Description of the auditory stimuli paradigms.

- *Subject’s own name (SON) paradigm* (Figure 2A)

Seven first names were selected for each subject (patient or healthy): the subject’s own first name (SON) and 6 other unfamiliar first names (OFNs). Before the experiment, the 6 OFNs were selected from a list of 18 disyllabic first names (nine masculine and nine feminine) of similar high frequency of occurrence in France (higher than 150 000 since 1900).^1^ The full list of first names was presented to the patient (or their relative), or to the healthy subjects, in order to exclude familiar first names (i.e., close relative’s name). The mean duration of the first names was 1.05 ± 0.05 sec. For each subject, 12 sequences of 42 names (1 SON + 6 OFNs presented 6 times in a pseudo-random order) were created. All first names were presented with the same probability (1/7 ≈ 14.3% for each name). The inter-stimulus interval (ISI) ranged from 400 to 500 msec. Each sequence was spoken with 1 voice randomly selected from 3. The duration of a sequence of 42 names was approximately 60 seconds. Each sequence was separated by 4 seconds. The duration of this experiment was approximately 12 minutes.

- *Semantic and Lexical Priming (SLP) paradigm* (Figure 2B)

This paradigm consisted of passively listening to pairs of items (words and/or pseudoword): a prime (word) following by a target (word or pseudoword). All stimuli were monosyllabic, with 3 to 6 letters and 2 to 5 phonemes. Word frequency of occurrence was greater than 2 per million. A set of 115 related congruent words pairs was created (based on the lexical database Lexique 3 available at [lexique.org](http://www.lexique.org)). The prime words were presented to 15 healthy volunteers (age mean (± SD): 37 (± 14) years, native French speakers, postgraduate) who had to give the most strongly semantically related target word they had in mind while listening to the prime. The 80 words pairs with the strongest cloze probability were selected for the experiment (mean (+/- SD) cloze probability = 0.55 (± 0.2)). Pairs of semantically unrelated words were created as following: their target words were selected from the target words of semantically related pairs. The prime words of unrelated pairs were pseudo randomly selected from the lexical database Lexique 3. Special attention was paid to avoid repetition and any possible association between prime and target unrelated words pairs. Thus, the targets words were the same in the related and unrelated pairs. One hundred and sixty monosyllabic pseudowords were also created from the target words by changing, adding or deleting 1 to 3 letters. A pseudoword is a succession of letters that is orthographically legal, can be pronounced, but has no meaning (e.g., *rande* or *guse*).^2^ The numbers of letters and phonemes were comparable between the target words and the pseudowords (p=0.93 and p=1 respectively). Finally, 80 pairs of related words, 80 pairs of unrelated words, and 160 pairs of word-pseudoword were created. The full lists of words pairs and word-pseudoword pairs are available on request. The entire protocol consisted of 1 block containing all pairs distributed in a pseudo-random order avoiding more than 3 successive repetitions of the same category of pairs. The ISIs between words in a pair was fixed at 400 msec. The ISI between pairs was variable and lasted from 1500 to 2000 msec, in random steps of 100 msec. A 60 seconds pause was introduced in the middle of the protocol to allow subjects to briefly relax. The duration of this experiment was approximately 21 minutes.

|  | **Overall population**  (n=48) | **DEL -**  (n=28) | **DEL +**  (n=20) | **p-value** |
| --- | --- | --- | --- | --- |
| Age (years)  Sex: male  BMI (Kg.m^-2^)  Level of education  Not graduate  Graduate  Post graduate | 65 [55 – 71.5]  42 (87.5%)  27.8 [25.5 – 30.8]  34 (70.8%)  7 (14.6%)  7 (14.6%) | 64.5 [56.5 – 71]  23 (82%)  27.7 [25.4 – 30.9]  16 (57.1%)  6 (21.4%)  6 (21.4%) | 66.5 [54 – 72.5]  19 (95%)  28.6 [26.1 – 30.8]  18 (90%)  1 (5%)  1 (5%) | 0.83  0.38  0.65  **0.02** |
| **Past Medical History**  Neurological medical history  Stroke / Epilepsy / Aneurysm / Migraine  Anxious depression  Cardiovascular diseases  Respiratory disease  Chronic renal disease  Malignancies  Immunodeficiency  Diabetes  Chronic liver disease  Alcohol abuse  Obesity (BMI ≥ 30 Kg.m^-2^)  Charlson Comorbidity Index  ADL (/6)  IADL (/8) | 6 (12.5%)  3 / 1 / 1 / 1  7 (14.6%)  28 (58.3%)  11 (22.9%)  3 (6.3%)  4 (8.3%)  3 (6.3%)  23 (47.9%)  1 (2.1%)  3 (6.3%)  15 (31.3%)  3 [1.5 – 4.5]  6 [6 – 6]  8 [8 – 8] | 4 (14.3%)  2 / 0 / 1 / 1  4 (14.3%)  17 (60.7%)  7 (25%)  2 (7.1%)  3 (10.7%)  1 (3.6%)  12 (42.9%)  0 (0%)  0 (0%)  8 (28.6%)  3 [2 – 4]  6 [6 – 6]  8 [8 – 8] | 2 (10%)  1 / 1 / 0 / 0  3 (15%)  11 (55%)  4 (20%)  1 (5%)  1 (5%)  2 (10%)  11 (55%)  1 (5%)  3 (15%)  7 (35%)  3 [1 – 6]  6 [6 – 6]  8 [8 – 8] | 1  1  0.77  0.74  1  0.63  0.56  0.56  0.42  *0.07*  0.76  0.42  0.40  0.25 |
| Positive SARS-CoV-2 RT-PCR  Time from symptoms onset to ICU admission  Time from positive RT-PCR to ICU admission | 48 (100%)  8 [6 – 10]  6 [2 – 8] | 28 (100%)  8 [6.5 – 10]  6 [3-8] | 20 (100%)  8 [6 – 10]  4.5 [1.5 – 8] | -  0.88  0.28 |
| **ICU stay**  SAPS II  SOFA  PaO2/FiO2 on admission (mmHg)  Mechanical Ventilation  Invasive / duration (days)  Non-Invasive / duration (days)  High Flow Oxygen Therapy / duration (days)  Prone positioning (awake or sedated)  Neuromuscular blocking agents / duration (days)  Sedative treatments  Midazolam  Duration (days)  Propofol  Duration (days)  Opioids  Duration (days)  Ketamine  Duration (days)  Alpha-2 agonists  Duration (days)  Vasopressors  Antiviral treatments  Lopinavir + Ritonavir or Remdesivir  Tocilizumab  Steroids | 37 [30.5 – 44]  4.5 [4 – 6.5]  90 [78.5 – 114]  27 (56.3%) / 17 [8 – 27.2]  38 (79.2%) / 5 [3 – 9]  47 (97.9%) / 5 [3 – 7]  34 (70.8%)  26 (54.2%) / 6.5 [3 – 12]  26 (54.2%)  11.5 [6 – 20]  23 (47.9%)  9 [4 – 13]  27 (56.3%)  14 [7 – 23.7]  12 (25%)  5 [2 – 11.5]  25 (52.1%)  7 [4 – 12]  21 (43.8%)  4 (8.3%)  1 (2.1%)  45 (93.8%) | 35 [30.5 – 42]  4 [3.5 – 5]  96 [83.5 – 127]  9 (32%) / 17 [6.7 – 33.2]  20 (71%) / 5 [4 – 9.5]  28 (100%) / 5 [3.5 – 8.5]  19 (67.9%)  8 (28.6%) / 8.5 [2.5 – 14]  8 (28.6%)  10.5 [4 – 23.5]  7 (25%)  11 [4.75 – 14.5]  9 (32.1%)  10 [5 – 25.75]  3 (10.7%)  10 [4 – 19]  7 (25%)  4 [4 – 6.5]  8 (28.6%)  1 (3.6%)  1 (3.6%)  27 (96.4%) | 40 [30.5 – 49.5]  5 [4 – 8]  80 [71.5 – 107.5]  18 (90%) / 17 [8 – 25]  18 (90%) / 4 [2 – 6]  19 (95%) / 4 [2 – 6]  15 (75%)  18 (90%) / 6 [3 – 11]  18 (90%)  12 [7 – 17]  16 (80%)  8.5 [3 – 13]  18 (90%)  16 [8 – 23]  9 (45%)  5 [2 – 8.5]  18 (90%)  7.5 [4 – 12]  13 (65%)  3 (15%)  0 (0%)  18 (90%) | 0.20  0.13  **0.02**  **<0.0001**  0.16  0.42  0.75  **<0.0001**  **<0.0001**  **<0.001**  **<0.0001**  **0.016**  **<0.0001**  **0.019**  0.29  1  0.56 |
| Length of stay (days)  ICU  Hospital  Family visitation (in person or virtual) | 13 [6.2 – 29]  24 [13.5 – 37]  43 (89.6%) | 8 [5 – 15]  15 [11.5 – 30]  26 (92.9%) | 21.5 [13.5 – 39]  35 [26.5 – 81.7]  17 (85%) | **<0.001**  **0.003**  0.64 |

*Supplementary Table 1***. Clinical and demographic characteristics.** Data are expressed as median [IQR] or n (%) as appropriate. *Abbreviations*: DEL -: COVID-19 patients without delirium; DEL +: COVID-19 patients with delirium; BMI: body mass index; ADL: Activities of daily living; IADL: Instrumental activities of daily living; RT-PCR: reverse transcriptase polymerase chain reaction; SAPS II: simplified acute physiology score II; SOFA: sequential organ failure assessment; ICU: intensive care unit.

| **Cerebral dysfunction measures** | **DEL + patients**  (n=20) |
| --- | --- |
| Subtypes of delirium  Hypoactive  Hyperactive  Mixed | 8 (40%)  6 (30%)  6 (30%) |
| Time from ICU admission to first clinical signs of delirium (days)  Delirium duration (days)  Coma duration (days)  Acute cerebral dysfunction duration (days) | 8 [3.5 – 20]  7 [4.5 – 9.5]  11.5 [6.5 – 20]  17.5 [13.5 – 30.5] |
| CAM-ICU 7 | 5 [4 – 5.5] |
| Length of stay in the ICU (days)  Acute cerebral dysfunction-free days | 21.5 [13.5 – 39]  3.5 [1 – 9.5] |
| Daily Hazard of Delirium  Acute cerebral dysfunction duration / length of stay | 61% [51 – 85.5]  81.5% [74 – 92.5] |
| Delirium at ICU discharge | 13 (65%) |

*Supplementary Table 2.* **Cerebral dysfunction characteristics.** Data are expressed as median [IQR] or n (%). Delirium was defined as a positive Confusion Assessment Method for the ICU (CAM-ICU)^3^ in the patient medical record during their ICU coronavirus disease 2019 stay. Coma was defined by Richmond Agitation Sedation Scale (RASS) score of –4 or –5. Hypoactive delirium was defined by RASS of –1 to –3 with positive CAM-ICU. Hyperactive delirium was defined by a RASS score of +1 to +3 with positive CAM-ICU.^4^ Delirium severity was measured using the CAM-ICU-7,^5^ a seven-point rating scale (0-7), derived from the RASS and the CAM-ICU. The CAM-ICU 7 score ranges from 0 to 7; categorized as 0-2: no delirium, 3-5: mild to moderate delirium, and 6-7: severe delirium. Daily Hazard of Delirium: defined as number of days the patient was delirium positive divided by the number of days at risk for delirium. *Abbreviations*: DEL +: COVID-19 patients with delirium; ICU: intensive care unit; CAM-ICU 7: seven-point rating scale for the confusion assessment method for the ICU.

| **Patient** | **Age** (years) / **Sex** | **Delirium** | **CAM-ICU 7** | **Delirium Subtypes** | **Delirium Duration**  (days) | **ERP recording time**  (days after ICU admission) | **RASS** at the time of **ERP recording** | **Language processing** | | |
| --- | --- | --- | --- | --- | --- | --- | --- | --- | --- | --- |
|  |  |  |  |  |  |  |  | **SON paradigm** | **SLP paradigm** | |
|  |  |  |  |  |  |  |  | **Self-relevant word discrimination** | **Semantic incongruence detection** | **Lexical incongruence detection** |
| 1 | 50 / M | DEL + | 7 | Mixed | 3 | 15 | -2 | No | **Yes** | No |
| 2 | 59 / M | DEL - | 0 | - | - | 9 | -1 | - | - | - |
| 3 | 54 /M | DEL + | 5 | Mixed | 8 | 20 | -1 | **Yes** | No | No |
| 4 | 48 / M | DEL + | 5 | Hyperactive | 5 | 20 | +2 | No | No | No |
| 5 | 77 / M | DEL + | 5 | Mixed | 4 | 2 | +2 | - | No | No |
| 6 | 78 / M | DEL - | 0 | - | - | 8 | 0 | **Yes** | No | No |
| 7 | 61 / M | DEL - | 0 | - | - | 1 | -1 | No | No | No |
| 8 | 75 / M | DEL - | 2 | - | - | 6 | -1 | No | No | No |
| 9 | 73 / F | DEL - | 0 | - | - | 4 | 0 | - | - | - |
| 10 | 78 / M | DEL - | 0 | - | - | 3 | -1 | No | No | No |
| 11 | 54 / M | DEL - | 0 | - | - | 4 | 0 | **Yes** | No | No |
| 12 | 65 / M | DEL - | 0 | - | - | 5 | -1 | - | - | - |
| 13 | 64 / M | DEL + | 6 | Mixed | 8 | 10 | -1 | No | No | No |
| 14 | 55 / M | DEL - | 0 | - | - | 2 | 0 | No | No | No |
| 15 | 75 / M | DEL + | 3 | Hypoactive | 3 | 10 | 0 | No | No | No |
| 16 | 69 / F | DEL - | 0 | - | - | 7 | 0 | - | - | - |
| 17 | 69 / M | DEL + | 4 | Hyperactive | 6 | 8 | +1 | No | No | No |
| 18 | 68 / M | DEL - | 0 | - | - | 1 | 0 | No | No | No |
| 19 | 54 / M | DEL + | 7 | Hypoactive | 13 | 22 | -2 | No | No | No |
| 20 | 65 / M | DEL - | 0 | - | - | 1 | 0 | No | No | No |
| 21 | 58 / M | DEL - | 1 | - | - | 23 | -2 | **Yes** | **Yes** | No |
| 22 | 66 / M | DEL + | 5 | Hyperactive | 18 | 23 | +1 | No | No | No |
| 23 | 77 / M | DEL + | 5 | Mixed | 29 | 35 | -1 | No | No | No |
| 24 | 80 / M | DEL + | 4 | Hypoactive | 7 | 6 | 0 | No | No | **Yes** |
| 25 | 68 / M | DEL - | 0 | - | - | 8 | +1 | - | - | - |
| 26 | 81 / F | DEL - | 0 | - | - | 2 | 0 | **Yes** | **Yes** | No |
| 27 | 58 / M | DEL + | 5 | Hypoactive | 11 | 45 | -1 | No | No | No |
| 28 | 66 / M | DEL - | 1 | - | - | 5 | 0 | - | - | - |
| 29 | 70 / M | DEL + | 7 | Hyperactive | 7 | 11 | +2 | No | No | No |
| 30 | 66 / M | DEL - | 0 | - | - | 49 | 0 | No | No | No |
| 31 | 58 / M | DEL - | 0 | - | - | 3 | -1 | **Yes** | No | No |
| 32 | 61 / M | DEL - | 1 | - | - | 7 | 0 | No | No | No |
| 33 | 60 / M | DEL + | 3 | Hypoactive | 4 | 33 | -1 | **Yes** | No | No |
| 34 | 64 / M | DEL - | 0 | - | - | 1 | 0 | No | No | No |
| 35 | 44 / M | DEL - | 0 | - | - | 11 | 0 | No | No | No |
| 36 | 67 / M | DEL + | 4 | Hypoactive | 5 | 20 | -1 | No | **Yes** | No |
| 37 | 54 / M | DEL - | 0 | - | - | 2 | 0 | No | No | No |
| 38 | 47 / M | DEL + | 5 | Hyperactive | 6 | 16 | +1 | No | - | - |
| 39 | 73 / M | DEL - | 0 | - | - | 13 | -1 | **Yes** | No | **Yes** |
| 40 | 59 /F | DEL - | 0 | - | - | 7 | 0 | No | No | No |
| 41 | 73 / M | DEL - | 0 | - | - | 2 | 0 | **Yes** | No | No |
| 42 | 50 / M | DEL + | 4 | Hyperactive | 9 | 37 | +1 | No | No | No |
| 43 | 37 / M | DEL - | 0 | - | - | 4 | 0 | **Yes** | No | **Yes** |
| 44 | 77 / M | DEL + | 4 | Hypoactive | 7 | 30 | -1 | No | No | No |
| 45 | 53 / M | DEL - | 0 | - | - | 3 | -1 | No | **Yes** | **Yes** |
| 46 | 55 / F | DEL - | 0 | - | - | 38 | 0 | No | No | No |
| 47 | 70 / M | DEL + | 6 | Mixed | 10 | 38 | -1 | No | No | No |
| 48 | 67 / F | DEL + | 4 | Hypoactive | 3 | 8 | -1 | No | No | No |

*Supplementary Table 3.* **Individual electrophysiological signatures of central language processing.** An individual effect was deemed present if and only if there was at least one cluster of significant effects (spatial-temporal clustering, p-cluster ≤ 0.01, p ≤ 0.05 at each sample) corresponding to a typical event-related potential (ERP) within the time window of interest identified at the group level (grand average). *Abbreviations*: M: male; F: female; CAM-ICU 7: seven-point rating scale for the confusion assessment method for the ICU; ICU: intensive care unit; SON: subject’s own name paradigm; SLP: semantic and lexical priming; DEL +: delirium positive; DEL -: delirium negative; ERP: event-related potentials; RASS: Richmond agitation and sedation scale

| **Patient** | **Age** (years) **/ Sex** | **GROUP** | **Prominent frequency background activity** (Hz) | **Asymmetry in frequency / voltage** | **Epileptiform discharges** | **Cyclic Alternating Pattern of Encephalopathy (CAPE)** | **Others** |
| --- | --- | --- | --- | --- | --- | --- | --- |
| 1 | 50 / M | DEL + | delta | no / no | no |  | long delta activities periods 1-2 Hz (bilateral) alternating with shorter periods of diffuse theta activity 5Hz. |
| 3 | 54 / M | DEL + | 8 | no / no | no | no | no |
| 4 | 48 / M | DEL + | 9 | no / no | no | no | no |
| 5 | 77 / M | DEL + | 6 | no / no | no | no | short periods with faster background activity around 7 Hz and some diffuse bilateral beta activities |
| 6 | 78 / M | DEL - | 8 | no / no | no | no | short bursts of beta activities, diffuse but prominent in frontal |
| 7 | 61 / M | DEL - | 8 | no / no | no | no | short bursts of beta activities, diffuse but prominent in frontal |
| 8 | 75 / M | DEL - | 7 | no / no | no | no | no |
| 10 | 78 / M | DEL - | 8 | no / no | no | no | low amplitude |
| 11 | 54 / M | DEL - | 8 | no / no | no | no | low amplitude, diffuse beta activities |
| 12 | 65 / M | DEL - | 6.5 | no / no | no | no | dermogram |
| 13 | 64 / M | DEL + | 6 | no / no | no | no | no |
| 14 | 55 / M | DEL - | 7 | no / no | no | no | no |
| 15 | 75 / M | DEL + | 7 | no / no | no | no | no |
| 17 | 69 / M | DEL + | 7 | no / no | no | no | few short bursts of bilateral diffuse beta activity |
| 18 | 68 / M | DEL - | 8.5 | no / no | no | no | no |
| 19 | 54 / M | DEL + | 7.5 | no / no | no | no | artefact T6 |
| 20 | 65 / M | DEL - | delta/theta, 4.5 | no / no | no | no | long periods of delta activity 4 Hz alternating with shorter periods about 7Hz. |
| 21 | 58 / M | DEL - | 8 | no / no | no | no | few short bursts 1 sec delta/theta frontal bilateral |
| 22 | 66 / M | DEL + | 6 | no / no | no | no | no (dermogram artefacts only) |
| 23 | 77 / M | DEL + | 5.5 | no / no | no | no | no |
| 24 | 80 / M | DEL + | 7 | no / no | no |  | few frontal intermittent delta rhythmic activities during 1 to 5 sec |
| 26 | 81 / F | DEL - | 8.5 | no / no | no | no | no (artefact in Cz that looks like a spike). |
| 27 | 58 / M | DEL + | 6 | no / no | no | no | seems asymmetric but due to noise/artefact on right hemisphere |
| 30 | 66 / M | DEL - | 8 | no / no | no | no | low amplitude |
| 31 | 58 / M | DEL - | 7 | no / no | no | no | low amplitude |
| 32 | 61 / M | DEL - | 7 | no / no | no | no | few short 1 sec delta burst frontal bilateral activities |
| 33 | 60 / M | DEL + | 8 | no / no | no | no | no |
| 35 | 44 / M | DEL - | 7 | no / no | no | no | no |
| 36 | 67 / M | DEL + | 6 | no / no | no | no | no |
| 38 | 47 / M | DEL + | 7 | no / no | no | no | low amplitude |
| 44 | 77 / M | DEL + | 6.5 | no / no | no | no | no |
| 48 | 67 / F | DEL + | 7 | no / no | no | no | no |

*Supplementary Table 4.* **Individuals results of standard EEG.** Standard EEG (10 +/- 3 min) was recorded at rest (‘passive’ condition) immediately before auditory stimuli presentation (‘active’ condition, corresponding to lexical/semantic and self-centered stimuli). This electrophysiological data was rated according to American Clinical Neurophysiology Society 2021 guidelines.^6^

|  | **P300 (Subject’s Own Name)** | | *p-value* |
| --- | --- | --- | --- |
|  | **+** (n=10) | **-** (n=31) |  |
| **ICU LOS** | 8 [5 – 22.25] | 16 [8 – 35.75] | 0.27 |
| **Hospital LOS** | 34.5 [11 – 42] | 27 [13.75 – 39.25] | 0.88 |

|  | **N400 (Semantic and/or Lexical Priming)** | | *p-value* |
| --- | --- | --- | --- |
|  | **+** (n=8) | **-** (n=33) |  |
| **ICU LOS** | 8 [5.25 – 26.75] | 15 [6.5 – 36] | 0.39 |
| **Hospital LOS** | 21 [10 – 27] | 31 [14.5 – 44] | 0.10 |

*Supplementary Table 5*. **Relationship between patients ICU and hospital length of stay and auditory ERPs.** Data are expressed as median [IQR] and were compared using the non-parametric Mann- Whitney U test. Abbreviations: ICU=intensive care unit; LOS=length of stay; (+) = ERP preservation; (-) = ERP abolition

**REFERENCES**

1. Perrin F, Schnakers C, Schabus M, et al. Brain response to one's own name in vegetative state, minimally conscious state, and locked-in syndrome. *Arch Neurol*. Apr 2006;63(4):562-9. doi:10.1001/archneur.63.4.562

2. Laszlo S, Federmeier KD. A Beautiful Day in the Neighborhood: An Event-Related Potential Study of Lexical Relationships and Prediction in Context. *J Mem Lang*. Oct 1 2009;61(3):326-338. doi:10.1016/j.jml.2009.06.004

3. Ely EW, Inouye SK, Bernard GR, et al. Delirium in mechanically ventilated patients: validity and reliability of the confusion assessment method for the intensive care unit (CAM-ICU). *JAMA*. Dec 5 2001;286(21):2703-10. doi:10.1001/jama.286.21.2703

4. Robinson TN, Raeburn CD, Tran ZV, Brenner LA, Moss M. Motor subtypes of postoperative delirium in older adults. *Arch Surg*. Mar 2011;146(3):295-300. doi:10.1001/archsurg.2011.14

5. Khan BA, Perkins AJ, Gao S, et al. The Confusion Assessment Method for the ICU-7 Delirium Severity Scale: A Novel Delirium Severity Instrument for Use in the ICU. *Crit Care Med*. May 2017;45(5):851-857. doi:10.1097/CCM.0000000000002368

6. Hirsch LJ, Fong MWK, Leitinger M, et al. American Clinical Neurophysiology Society's Standardized Critical Care EEG Terminology: 2021 Version. *J Clin Neurophysiol*. Jan 1 2021;38(1):1-29. doi:10.1097/WNP.0000000000000806
